# Influenza Virus and Vaccination

**DOI:** 10.3390/pathogens9030220

**Published:** 2020-03-17

**Authors:** Aitor Nogales, Marta L. DeDiego

**Affiliations:** 1 Center for Animal Health Research, INIA-CISA, 28130 Madrid, Spain; 2 Department of Molecular and Cell Biology, Centro Nacional de Biotecnología (CNB-CSIC), Campus Universidad Autónoma de Madrid, 28049 Madrid, Spain

**Keywords:** Influenza virus, influenza vaccine, vaccination, pandemic, immune response, innate immunity, adaptive immunity, universal vaccines

## Abstract

Influenza virus infections represent a serious public health problem causing contagious respiratory disease and substantial morbidity and mortality in humans, resulting in a considerable economic burden worldwide. Notably, the number of deaths due to influenza exceeds that of any other known pathogen. Moreover, influenza infections can differ in their intensity, from mild respiratory disease to pneumonia, which can lead to death. Articles in this Special Issue have addressed different aspects of influenza in human health, and the advances in influenza research leading to the development of better therapeutics and vaccination strategies, with a special focus on the study of factors associated with innate or adaptive immune responses to influenza vaccination and/or infection.

Sangster et al. provide a comprehensive picture of HA-specific antibody response to influenza virus infection, relevant for protecting the host against the infection. Moreover, the authors discuss the importance of the composition of an individual’s HA-reactive preexisting memory B cell (MBC), a population that can reflect the imprint of early-life HA exposure, and its role in determining the character of the HA-reactive antibody response. Furthermore, the authors suggest that antibodies resulting from preexisting MBC activation are important regulators of anti-HA antibody production, and play a role in the positive selection of germinal center B cells which are reactive to novel HA epitopes. The understanding of MBC competition, immunodominance hierarchies, and antibody regulation of B cell responses will help to improve influenza vaccine composition and administration approaches [1]. As reviewed by Jiong et al., traditional assays to study complex humoral responses after influenza infection or vaccination, such as HA-specific antibody responses, are limited in scope and too resource-intensive. However, multidimensional assays developed in recent years could overcome these problems by simultaneously measuring antibodies against a large panel of influenza HA proteins in a high throughput assay [2]. Misra and Nayak have highlighted the importance of vaccinating children and pregnant women against influenza, since influenza virus infection is responsible for significant morbidity and mortality in these populations. Disturbingly, the authors indicated that despite the benefits of the influenza vaccine, vaccination rates around the world remain well below targets. The constantly changing HA antigenicity of the influenza virus, along with the complexity of serological responses induced by influenza infections in the immune system, muddies efforts to interpret serology testing results or develop more effective vaccines [3]. In order to improve the safety of the live-attenuated influenza vaccine (LAIV) and make it available to a broader population (it is currently not recommended for children under the age of two, immunocompromised individuals, the elderly, and pregnant adults), Hilimire et al. have demonstrated that the influenza A virus master donor virus (MDV) A/Ann Arbor/6/60 H2N2 LAIV can inhibit host gene expression using both the PA-X and NS1 viral proteins. Furthermore, they show that by removing PA-X, the replication of the MDV LAIV is decreased in a mouse model, while maintaining full protective efficacy, demonstrating a broadly applicable strategy of tuning the amount of host antiviral responses induced by the IAV MDV for the development of improved and safer LAIVs [4].

Topham et al. discuss recent advances in an important branch of adaptive immune responses, the tissue resident memory (TRm) CD_8_ T cells, which comprise a cell population that forms in peripheral, nonlymphoid tissue after infection, and that do not recirculate into the bloodstream or other tissues. This cell population has been shown to be important against secondary encounters with a previously seen pathogen. However, many questions remain regarding our understanding of this unique cell subset and its role during influenza infections [5]. Previously, Sant’s laboratory has demonstrated that CD_4_ T cells specific for epitopes derived from HA are the most effective in providing help for the HA-specific B cell responses to infection and vaccination. In this special issue, Zackery et al. ask whether HA epitopes recognized by CD_4_ T cells in the primary response to infection are equally distributed across the HA protein. Using mice, their studies revealed that the HA-specific CD_4_ T cell epitopes cluster in two distinct regions of HA, which could be important in the development of universal vaccines against influenza [6].

Nogales and DeDiego discuss the importance of human genome polymorphisms for the susceptibility of some individuals to suffer more severe symptoms after influenza infections and for vaccine effectiveness. Notably, the knowledge and analysis of host genome variability will be a valuable tool with which to predict the outcomes of viral diseases and of prophylactic or therapeutic interventions, including vaccines and drugs [7].
